# Disease and Injury Trends following Heavy Rains in Western Japan in 2018

**DOI:** 10.31662/jmaj.2022-0122

**Published:** 2023-01-31

**Authors:** Chiaki Hashimoto, Takashi Yorifuji, Kazuharu Murakami, Shigeru Suganami

**Affiliations:** 1 Department of Epidemiology, Graduate School of Medicine, Density and Pharmaceutical Sciences Okayama University, Okayama, Japan; 2 Mabi Memorial Hospital, Okayama, Japan; 3 Association of Medical Doctors of Asia, Okayama, Japan

**Keywords:** Common disease, communicable disease control, disaster, flood, heavy rains, Okayama

## Abstract

**Introduction:**

Torrential rains occurred in Okayama in western Japan in July 2018, forcing local residents to evacuate. Few studies have reported early-phase disease and injury trends among patients following torrential rains. Thus, in this study, we assessed the illness and injury trends among patients who visited temporary medical facilities located in the areas affected by the 2018 torrential rains; these facilities opened 10 d after the disaster.

**Methods:**

We evaluated the trends among patients who visited a medical clinic located in the area in western Japan affected by heavy rains in 2018. We reviewed medical charts related to 1,301 outpatient visits and conducted descriptive analyses.

**Results:**

More than half of the patients were over 60 years old. The patients experienced mild injuries (7.9% of total visits) as well as common diseases such as hypertensive diseases (30%), diabetes mellitus (7.8%), acute upper respiratory infections (5.4%), skin diseases (5.4%), and eye diseases (4.8%). Hypertensive diseases were the main cause of a visit in any week. Eye problems were the second-highest reason for a visit in the first week, but there was a relative decrease from the first to the third week. Additionally, the proportion of injuries and skin diseases increased from the first to the second week, from 7.9% to 11.1% for injuries, and from 3.9% to 6.7% for skin diseases.

**Conclusions:**

The types of diseases changed on a weekly basis. Older adults needed medical support for longer than other age groups. Prior preparedness such as earlier deployment of such temporary clinics can help mitigate the damage to the victims.

## Introduction

Health problems caused by disasters are diverse. In the case of earthquakes, for example, problems include bruises, cuts, fractures, and other injuries and crash syndrome ^(1), (2)^. Additionally, the subsequent evacuation is reported to worsen pre-existing conditions or increase the risk of myocardial infarction ^(3)^, cerebral infarction ^(4)^, and mental illness. Studies from the Great East Japan Earthquake demonstrated that there was an elevated risk of upper respiratory infections ^(5), (6)^, gastrointestinal disorders ^(7), (8)^, sleep disorders ^(9)^, mental distress ^(10)^, cognitive dysfunction ^(11)^, and nutritional deficiencies ^(12)^ among the victims. Flooding can also cause medical complications such as waterborne infections ^(13)^, hypothermia ^(14)^, wounds ^(14)^, respiratory infections ^(15)^, gastrointestinal infections ^(14)^, and mental illness ^(16)^. By contrast, however, there are few reports on the health effects of torrential rains ^(17), (18)^, which are common in Japan.

Torrential rains is defined by the Japan Meteorological Agency as a significant heavy rainfall event that results in a substantial disaster ^(19)^. Unlike the victims from earthquakes and flooding, the victims affected by torrential rains tend to remain in or near their own homes after the disaster because torrential rains do not always destroy their homes. While remaining close to home has some advantages such as facilitating the cleaning of the home, it also has disadvantages, which include victims having to live in water-damaged houses or getting injured from cleaning. Additionally, victims have few opportunities to receive medical care because medical facilities in the affected areas are sometimes shut down.

In this study, we aimed to assess the trends of illnesses and injuries among patients who visited temporary medical facilities located in the areas affected by the 2018 torrential rains in western Japan. These facilities opened 10 d after the disaster. The findings of this study have implications for understanding medical needs in the event of future widespread torrential rains.

## Materials and Methods

### Study design

We employed a descriptive epidemiological study design for this study.

### Study area and participants

**Figure 1.**
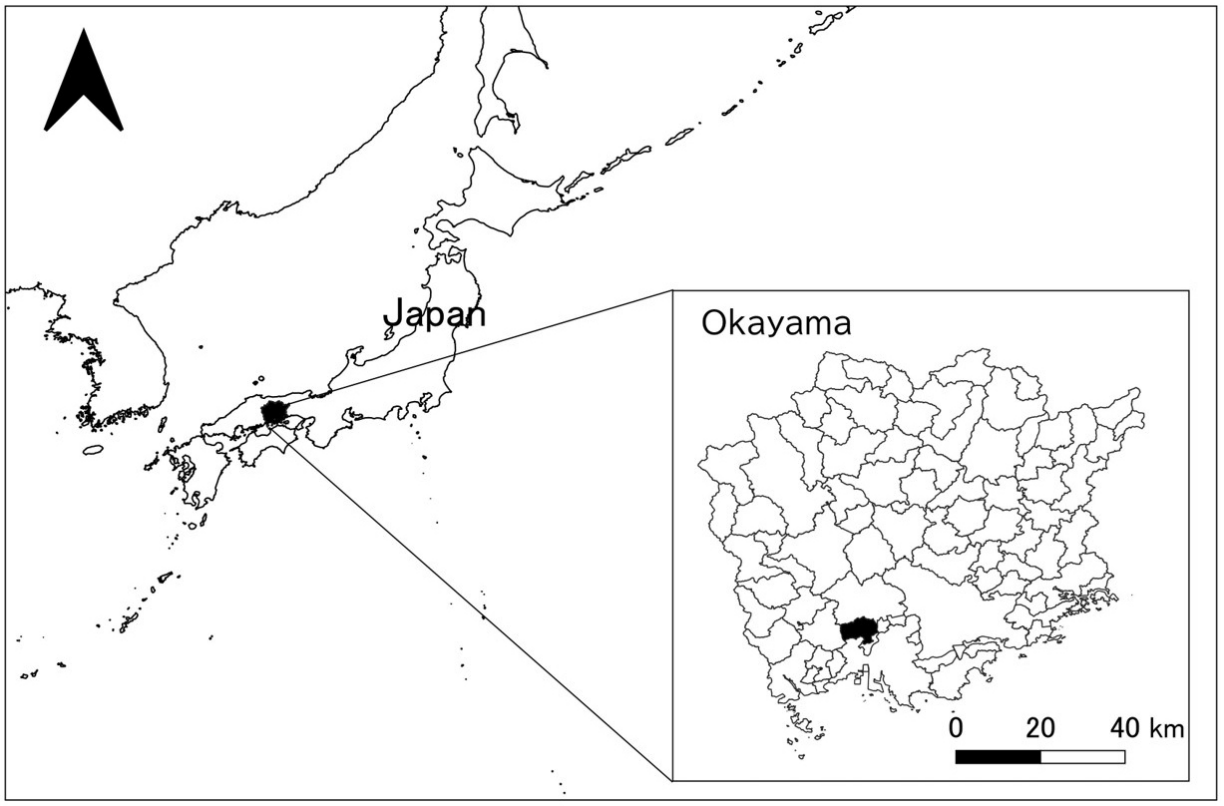
Location of Mabi-town, Kurashiki City, Okayama, Japan.

From late June to July 8, 2018, heavy rainfalls with devastating floods and landslides were widespread in southwestern Japan. In Okayama Prefecture, several levees broke, causing massive flooding damage mainly in Mabi-town, Kurashiki City (Figure 1).

The town of Mabi is located in the Okayama Prefecture in southwestern Japan. According to the late June 2018 census, the population of Mabi-town was 22,797, with 9,006 households ^(20)^, and 33.7% of the population were over 65 years of age. The torrential rains affected 27% of the town and flooded 1,200 hectares. The damage caused by the flood in Mabi has been estimated to include 52 deaths, 104 injuries, and more than 5,000 destroyed buildings (Kurashiki City summary as of October 12, 2018) ^(21)^.

Mabi-town had two hospitals, 10 clinics, and seven dental clinics before the torrential rains. After the disaster, all of them were flooded except for the psychiatric hospital, which was located on higher ground ^(21)^, making it impossible to continue medical services. Mabi Memorial Hospital, with 80 general beds, was the only general hospital in the area, but because it was located in the flooded area, it was also affected by the rains.

### Setting in the temporary clinic

Mabi Memorial Hospital, Kibi Medical Association, and the Association of Medical Doctors of Asia (AMDA), established a temporary clinic in a medical vehicle on the premises of Mabi Memorial Hospital 10 d after the disaster on July 18, 2018. A total of 1,301 outpatients visited the clinic between July 18 and August 14 of that year.

Medical services at the temporary clinic were provided under the Japanese health insurance system. For those affected above a certain level, the copayments and user fees for the National Health Insurance were postponed or exempted.

During the four weeks when the clinic was open, there were two doctors, eight nurses, three medical clerks, and three to five pharmacists and nonmedical volunteers available, depending on the situation in the team. The team first used a medical checkup van equipped with height and weight scales, X-rays, and echoes. The team could also use routine medical equipment (e.g., thermometers, sphygmomanometers, stethoscopes, flashlights, tongue depressors, pulse oximeters, urinalysis, and blood tests) in the van. After a week, the team moved the clinic to a prefabricated structure on the premises of the Mabi Memorial Hospital.

**Figure 2.**
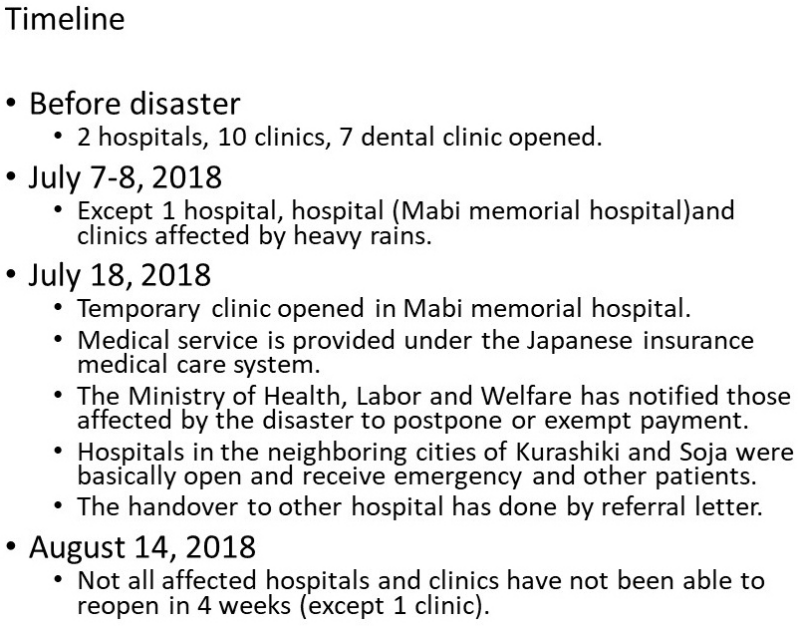
Timeline of medical service situation, Mabi-town and neighboring city.

As mentioned, Mabi-town had two hospitals (i.e., Mabi Memorial Hospital and one psychiatric hospital) and 10 clinics before the torrential rains, but only the psychiatric hospital could continue medical services after the disaster (Figure 2). Even though about four weeks had passed since the beginning of the torrential rains, only one clinic could reopen the facility. When further medical services were needed for the patients, referral letters were provided from the temporary clinic, and the patients visited hospitals in neighboring cities.

### Data collection

We reviewed the medical charts from the temporary clinic and collected patients’ data, including age, sex, physician diagnosis, and whether patients were prescribed drugs, administered intravenous fluids, or referred to hospitals. When there were two or more diagnoses related to the visits, we selected the primary diagnosis based on the principal reason for the visit or the prescribed medication. Finally, we classified each diagnosis according to the condensed list of disease causes from the Japanese Ministry of Health, Labour, and Welfare, which is based on the International Classification of Diseases, Tenth Revision ^(22)^.

### Analysis

We conducted descriptive analyses. First, we examined the distribution of the causes of disease and injury in total and separated by age category. We also checked changes in the distribution of disease and injury from the first to the fourth week. We finally evaluated the number of visits or days of visits according to age category: under 5 years old, 5-19 years old, 20-39 years old, 40-59 years old, 60-79 years old, and over 80 years old and over.

We used Stata SE, version 17, statistical software (Stata Corp., College Station, TX, USA) for all analyses. This study was approved by the Institutional Review Board of the Okayama University Graduate School of Medicine, Dentistry, and Pharmaceutical Sciences (No. 2008-047), which exempted requirement for informed consent because this was a retrospective study, and complete anonymity was ensured.

## Results

More than half of the patients were female, and approximately half of the patients were over 60 years old (Table 1). The team prescribed medications for over half of the patients, approximately 0.4% of patients needed intravenous fluid administration, and 2% were referred to hospitals.

**Table 1.** Demographic Characteristics of Patients Who Visited a Clinic Located at the Town of Mabi Memorial Hospital, Japan (Affected Area of the Western Japan’s Heavy Rain) from July 18 to August 15, 2018 (n = 1,301).

			n	%
Sex				
	Men		592	45.5
	Women		709	54.5
Age (years)				
	<5		11	0.9
	5-19		50	3.9
	20-39		69	5.3
	40-59		178	13.7
	60-79		716	55.0
	≥80		277	21.3
Prescription drug use			1,224	94.1
Intravenous fluid administration			5	0.4
Referral to hospitals			26	2.0

Table 2 shows the distribution of causes of diseases in total and separated by age category. The patients experienced mild injuries (7.9% of total visits) as well as common diseases such as hypertensive diseases (30%), diabetes mellitus (7.8%), acute upper respiratory infections (5.4%), skin diseases (5.4%), and eye diseases (4.8%). There were, however, differences in the types of disease according to age category. While skin problems, acute upper respiratory infections, and eye diseases were common among the young, hypertensive disease, diabetes mellitus, and musculoskeletal problems were more prevalent among older patients.

**Table 2.** Distribution of Diseases and Injuries among Patients, in Total and Separated by Age Category (n = 1,301).

	Total		<20 years		20-59 years		≥60 years	
	(n = 1301)		(n = 61)		(n = 247)		(n = 993)	
	n	%	n	%	n	%	n	%
Certain infectious and parasitic diseases
Intestinal infectious diseases	13	1.0	3	4.9	6	2.4	4	0.4
Viral infections characterized by skin and mucous membrane lesions	6	0.5	1	1.6	1	0.4	4	0.4
Mycoses	1	0.1	0	0.0	0	0.0	1	0.1
Other infectious diseases	3	0.2	0	0.0	3	1.2	0	0.0
Neoplasms								
Malignant neoplasms	4	0.3	0	0.0	1	0.4	3	0.3
Diseases of the blood and blood-forming organs								
Anemia	4	0.3	0	0.0	2	0.8	2	0.2
Endocrine, nutritional, and metabolic diseases								
Disorders of thyroid gland	18	1.4	0	0.0	2	0.8	16	1.6
Diabetes mellitus	101	7.8	0	0.0	14	5.7	87	8.8
Disorders of lipoprotein metabolism and other lipidemias	53	4.1	0	0.0	8	3.2	45	4.5
Endocrine, nutritional, and metabolic diseases	12	0.9	0	0.0	3	1.2	9	0.9
Mental and behavioral disorders
Mood affective disorders	3	0.2	0	0.0	3	1.2	0	0.9
Neurotic, stress-related, and somatoform disorders	8	0.6	0	0.0	1	0.4	7	0.7
Other mental and behavioral disorders	6	0.5	0	0.0	0	0.0	6	0.6
Diseases of the nervous system, including insomnia	34	2.6	0	0.0	10	4.1	24	2.4
Diseases of the eye and adnexa								
Cataract	4	0.3	0	0.0	0	0.0	4	0.4
Other diseases of the eye and adnexa	62	4.8	6	9.8	11	4.5	45	4.5
Diseases of the ear and mastoid process								
Diseases of middle ear and mastoid	2	0.2	2	3.3	0	0.0	0	0.0
Diseases of inner ear	3	0.2	0	0.0	0	0.0	3	0.3
Diseases of the circulatory system								
Hypertensive diseases	390	30.0	0	0.0	41	16.6	349	35.2
Ischemic heart diseases	21	1.6	0	0.0	1	0.4	20	2.0
Other heart diseases	15	1.2	0	0.0	4	1.6	11	1.1
Cerebral infarction	12	0.9	0	0.0	1	0.4	11	1.1
Other cerebrovascular diseases	1	0.1	0	0.0	0	0.0	1	0.1
Other diseases of the circulatory system	5	0.4	0	0.0	1	0.4	4	0.4
Diseases of the respiratory system								
Acute upper respiratory infections	70	5.4	14	23.0	27	10.9	29	2.9
Pneumonia	3	0.2	2	3.3	1	0.4	0	0.0
Bronchitis and chronic obstructive pulmonary disease	15	1.2	2	3.3	1	0.4	12	1.2
Asthma	17	1.3	4	6.6	7	2.8	6	0.6
Other diseases of the respiratory system	14	1.1	2	3.3	7	2.8	5	0.5
Disease of the digestive system								
Other disorders of teeth and supporting structures	25	1.9	3	4.9	3	1.2	19	1.9
Gastric and duodenal ulcer	6	0.5	0	0.0	1	0.4	5	0.5
Gastritis and duodenitis	18	1.4	0	0.0	3	1.2	15	1.5
Disease of liver	1	0.1	0	0.0	1	0.4	0	0.0
Other diseases of the digestive system, including constipation	35	3.7	1	1.6	6	2.4	28	2.8
Diseases of the skin and subcutaneous tissue	70	5.4	11	18.0	13	5.3	46	4.6
Diseases of the musculoskeletal system and connective tissue, including stiff shoulder and low back pain								
Inflammatory polyarthropathies	11	0.9	0	0.0	4	1.6	7	0.7
Spondylopathies	45	3.5	0	0.0	7	2.8	38	3.8
Disorders of bone density and structure	12	0.9	0	0.0	0	0.0	12	1.2
Other disorders of the musculoskeletal system and connective tissue	32	2.5	1	1.6	5	2.0	26	2.6
Diseases of the genitourinary system								
Other disorders of the genitourinary system	17	1.3	0	0.0	1	0.4	16	1.6
Symptoms, signs, and abnormal clinical and laboratory findings, not elsewhere classified	25	1.9	4	6.6	6	2.4	15	1.5
Injury, poisoning, and certain other consequences of external causes	0	0.0	0	0.0	0	0.0	0	0.0
Fracture	1	0.1	0	0.0	0	0.0	1	0.1
Other factors influencing health status and contract with health services	103	7.9	5	8.2	41	16.6	57	5.7

Note: We used a conducted list of causes of disease based on the International Classification of Diseases, Tenth Revision and Simple Classification of Causes of Death. We omitted the unknown age category, owing to the small number of cases.

**Figure 3.**
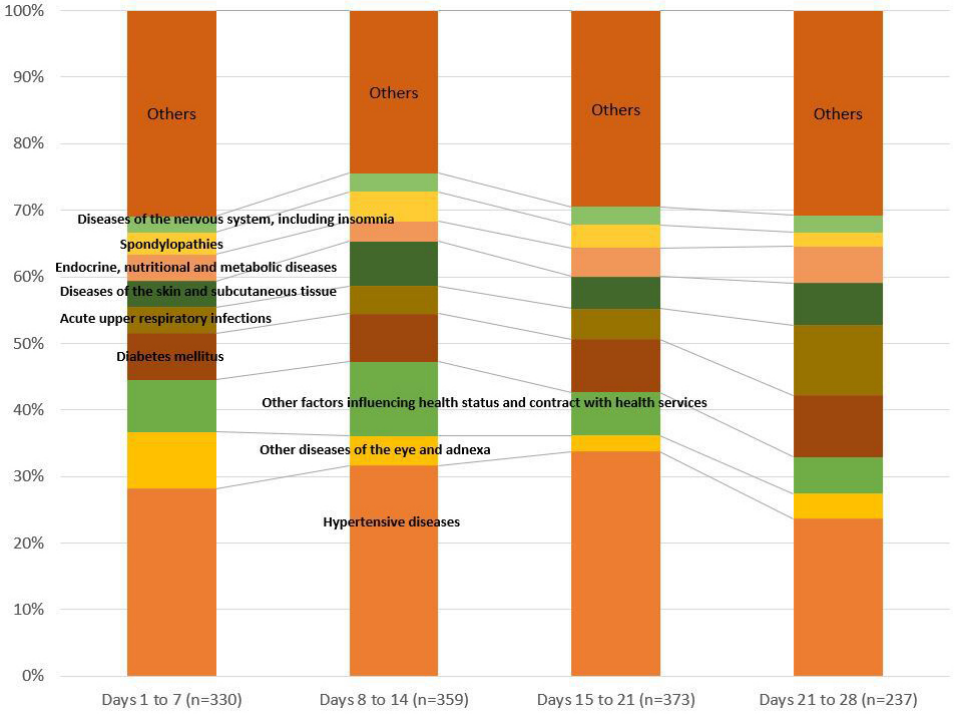
Distribution of diseases and injuries according to visit days at the clinic after the first reopen day on July 18.

Changes in the distribution of diseases and injuries from the first to the fourth week are shown in Figure 3. Hypertensive diseases were the main cause of a visit in any week. In the first week after the temporary clinic opened (i.e., during the period July 18-25), there were 330 visits for all causes, with 28.2% for hypertensive diseases, 8.5% for eye diseases, and 7.9% for injuries. Eye problems were the second-highest reason for a visit in the first week, but there was a relative decrease from the first to the third week. Additionally, the proportion of injuries and skin diseases increased from the first to the second week, from 7.9% to 11.1% for injuries, and from 3.9% to 6.7% for skin diseases. The proportion of acute upper respiratory infections also increased from the first to the fourth week (3.9% to 10.6%)

When we evaluated the number of visits or visit days according to age category, patients aged over 40 years tended to have more visits or continued visits in the latter weeks compared with that of younger patients (Table 3). For example, 1.4% of the patients aged over 80 years and over visited more than five times, while patients aged under 20 years tended to visit only once or twice.

**Table 3.** Number of Visits and Days of Visits after Reopening the Clinic on July 18, as Separated by Age Category (n = 1301).

	Total		<5		5-19		20-39		40-59		60-79		≥80	
	(n = 1,301)		(n = 11)		(n = 50)		(n = 69)		(n = 178)		(n = 716)		(n = 277)	
	n	%	n	%	n	%	n	%	n	%	n	%	n	%
Number of visits														
One	1115	85.7	11	100.0	45	94.2	65	94.2	154	86.5	617	86.2	223	80.5
Two	149	11.5	0	0.0	5	4.4	3	4.4	19	10.7	85	11.9	37	13.4
Three	25	1.9	0	0.0	0	1.5	1	1.5	4	2.3	11	1.5	9	3.3
Four	8	0.6	0	0.0	0	0.0	0	0.0	1	0.6	3	0.4	4	1.4
≥Five	4	0.3	0	0.0	0	0.0	0	0.0	0	0.0	0	0.0	4	1.4
Days of visits after the reopen the clinic														
Day 1-7	330	25.4	3	27.3	13	26.0	13	26.0	42	23.6	193	27.0	66	23.8
Day 8-14	360	27.7	2	18.2	17	34.0	22	34.0	47	26.4	199	27.8	73	26.4
Day 15-21	373	28.7	2	18.2	14	28.0	17	28.0	58	32.6	188	26.3	94	33.9
Day 22-28	237	18.2	4	36.4	6	12.0	17	12.0	31	17.4	135	18.9	44	15.9
Unknown	1	0.1	0	0.0	0	0.0	0	0.0	0	0.0	1	0.1	0	0.0

## Discussion

In this study, we evaluated the trends of injuries and illnesses among patients who visited a temporary clinic established in areas affected by torrential rains in western Japan. Patients suffered from common illnesses and injuries, and the types of illnesses varied from week to week. In addition to injuries, major illnesses included hypertension, diabetes, acute upper respiratory infection, skin diseases, and eye diseases. Older adults required medical assistance longer than other age groups.

Injuries were the reason for a large percentage of visits to the health clinic during the study period. The causes of the injuries included those directly related to the disaster or those related to cleaning up when returning home.

The most common reasons for seeking medical attention were chronic diseases such as hypertension, diabetes, and dyslipidemia. In times of disaster, the spread of damage and the shortage of medicines and medical equipment cause the medical system to collapse. The patients’ ability to manage themselves deteriorates, and diseases that were under control in normal times worsen. In our study, many patients developed hypertension. Three main factors contributed to this: medications being washed away; excessive salt intake from the consumption of high-sodium emergency supplies; and increased stress factors. Several studies ^(4), (23)^ have reported an increased risk of heart disease after an earthquake, suggesting the need for the proper management of hypertension.

The prevalence of eye diseases was 8.5% in the first week, making it the second most common condition after hypertension. Floods and heavy rains can carry water contaminated with bacteria, parasites, and viruses over large areas, polluting the environment and contaminating water sources. Previous studies on floods have shown that they increase the incidence of waterborne ^(13)^ and postflood epidemics as well as eye diseases ^(24)^. The increase in eye diseases due to heavy rains has been attributed to two factors. First, flood victims often remain in the flood area, which often has poor sanitation. Second, when the water recedes from flooded areas, the land dries, and contaminated soil is more likely to be flung into the air and, consequently, come into contact with patients’ eyes. In this survey, it was shown that not only evacuees and victims whose homes were damaged sought treatment for eye diseases but also volunteers working in the affected areas.

The incidence of infectious diseases, especially upper respiratory infections, increased along with the number of weeks. In the past survey for the Kumamoto earthquake ^(25)^, the incidence of upper respiratory infections increased from the first to the second week. This suggests that evacuation may be related to the increase in the incidence of these infections. The rate of consultations for skin diseases increased gradually. As has been the case in previous postearthquake evacuation shelter surveys, it is difficult to secure clean water after heavy rains. Older adults, especially those who have difficulty moving their bodies, seem to have difficulty keeping clean. Additionally, calcium hydroxide was used for postflood disinfection in Kurashiki City, including Mabi-town, which possibly affected the increased morbidity caused by eye diseases, skin diseases, and upper respiratory tract infections ^(26)^.

In other countries, diseases that commonly occur during the acute phase of disasters include skin diseases, eye diseases, and respiratory diseases. On the other hand, vector-borne infectious diseases such as leptospirosis and malaria are frequently reported overseas, more so than in Japan.

The torrential rains discussed in this case were compared to the tsunami floods and the Great East Japan Earthquake that occurred in Japan in the past. Similarities include the fact that many patients were exposed to dried sludge, and a certain percentage developed respiratory problems. Another similarity is the situation where evacuees were crowded together in shelters for a long period of time.

In the case of the Great East Japan Earthquake, it is said that dried sludge pushed up the incidence of respiratory diseases and respiratory allergies ^(27)^, and it is reported that the number of hospitalizations due to respiratory diseases increased from that before the disaster.

In the recent torrential rains in western Japan, acute upper respiratory tract infections also increased from the first to fourth week after the disaster. Although the occurrence of respiratory tract infections peaked early in the Great East Japan Earthquake as well, most of the previous studies conducted in hospitals and evacuation centers from 10 d to 2 months after the earthquake focused on infectious and respiratory diseases and did not simultaneously investigate chronic diseases such as hypertension, preventing accurate comparisons.

If previous studies had included chronic diseases, seawater sludge could affect respiratory diseases earlier than river sludge, as the incidence of acute upper respiratory tract infections increased slowly on a weekly basis during the recent heavy rain disaster.

Studies on the Great East Japan Earthquake and other earthquakes have shown that the victims often suffered from mental health problems. The percentage of victims experiencing mental health problems was not apparent in the present study. This could be because the target population included not only the victims in evacuation centers but also those visiting from their homes and volunteers.

Older adults needed medical assistance for longer than other age groups. This is consistent with previous findings, wherein older adults are more susceptible to stress from earthquakes as compared to other age groups.

One strength of this study is that we collected information on patients’ illnesses and injuries directly from a hospital rather than from the evacuees at shelters. Additionally, medical records were collected 10 d after the heavy rains. Furthermore, medical decisions were based on the diagnoses of doctors.

Several limitations deserve mention. First, we could not take into account weekly changes in the denominator, which could have been caused by the changing numbers of evacuees living close to the temporary clinic. It is possible that some people, mainly those of working age, left the affected area. This may partly explain the declining number of total patients, especially younger patients, from the first week to the fourth week. Second, all of the patients who needed some medical care in the affected region may not have visited the temporary clinic. Third, there may be a problem of generalization. Caution will be needed when generalizing the findings to other evacuation centers, to other clinics, to evacuation centers in other countries with different sanitary conditions, or to other affected clinics.

In conclusion, in this study, we identified minor injuries and common illnesses among patients who visited a temporary clinic located in an area affected by heavy rains. The types of illnesses varied weekly, and we found that older adults had a longer recuperation period than younger patients did. Future floods may be inevitable, but prior preparedness can help the earlier deployment of temporary clinics, mitigating damage to the victims.

## Article Information

### Conflicts of Interest

None

### Sources of Funding

This work was supported by the JSPS KAKENHI Grant Number JP20K1049902. The sponsor had no involvement in the study design, data collection, analysis, interpretation, manuscript writing, or decisions regarding publication submission.

### Acknowledgement

We appreciate the support of the medical staff at the temporary clinic, the people involved in running the clinic, Dr.Saori Kashima (Hiroshima University), Mr. Sumihiro Kunishige (Mabi Memorial Hospital), Mr. Toshiyuki Obata (Mabi Memorial Hospital), Ms. Saori Irie (Okayama University), and Ms. Yoko Oka (Okayama University). We are also extremely grateful for the help from the local residents. We thank Lucy McClellan, MIA, from Edanz (https://jp.edanz.com/ac) for editing a draft of this manuscript.

### Author Contributions

Chiaki Hashimoto analyzed the data and wrote the first draft. Takashi Yorifuji, Kazuharu Murakami, and Shigeru Suganami contributed to the design of the study, interpreted the data, and revised the manuscript. All authors read and approved the final manuscript.

### Approval by Institutional Review Board (IRB)

Approval for this study was obtained from the Institutional Review Boards of Okayama University (No. 2008-047).
